# Domestic dogs (*Canis familiaris*) grieve over the loss of a conspecific

**DOI:** 10.1038/s41598-022-05669-y

**Published:** 2022-02-24

**Authors:** Stefania Uccheddu, Lucia Ronconi, Mariangela Albertini, Stanley Coren, Gonçalo Da Graça Pereira, Loriana De Cataldo, Anouck Haverbeke, Daniel Simon Mills, Ludovica Pierantoni, Stefanie Riemer, Ines Testoni, Federica Pirrone

**Affiliations:** 1 San Marco Veterinary Clinic and Laboratory, 35130 Veggiano, Padua Italy; 2 Department of Philosophy, Sociology, Education and Applied Psychology, University of Padua, 35122 Padua, Italy; 3 Department of Veterinary Medicine and Animal Science, University of Milan (UNIMI), 26900 Lodi, Italy; 4 Department of Psychology, University of British Columbia, Vancouver, V5V 3K3 Canada; 5 Departamento de Medicina Veterinária, Universidade de Évora (EU), Évora, Portugal; 6 Escola Superior Agrária de Elvas (ESAE), Instituto Politécnico de Portalegre (IPP), 7350-092 Elvas, Portugal; 7 Vet Ethology, 3090 Overijse, Belgium; 8 Salto Research Group, Odisee University College, Hospitaalstraat 23, 9100 Sint-Niklaas, Belgium; 9 School of Life Sciences, University of Lincoln, Lincoln, LN6 7DL Lincolnshire UK; 10 Veterinary Behaviour & Consulting Services, CAN Training Centre, 80128 Naples, Italy; 11 Companion Animal Behaviour Group, Division of Animal Welfare, Vetsuisse Faculty, University of Bern, 3012 Bern, Switzerland

**Keywords:** Animal behaviour, Psychology

## Abstract

Behavioural reactions towards a dead conspecific have been observed rarely in wild canids and there is no documented scientific evidence of grief in pet dogs. A quantitative analysis of grief-related responses in both dogs and owners was conducted, using the validated online Mourning Dog Questionnaire. The survey was completed by 426 Italian adults who had owned at least two dogs, one of whom died while the other was still alive. This research aims to explore whether, how and what a dog may experience over the loss of a companion dog. Multiple logistic regression indicates that both a friendly or parental relationship between two dogs but also the fact that dogs used to share food and the owner’s grief and anger are principal predictors of negative behavioural changes. According to dog owners’ answers, the surviving dog after the death of the companion dog changed both in terms of activities (“playing”, “sleeping”, and “eating”) and emotions (fearfulness), which occurred as a function of the quality of the relationship between the two animals. By contrast, the time the two dogs had spent together had no effect on the behaviours of surviving dog. Owner perceptions about their dog’s reactions and emotions were not related to the memory or suffering of the event that tended to diminish over time. These findings indicate that a dog may show grief-related behavioural and emotional patterns when a close conspecific dies, with aspects of the latter possibly related to the owner’s emotional status.

## Introduction

Grief responses are widely reported in social species such as great apes, whales, dolphins, elephants and birds, which have been described to engage in death rituals^1^, including touching and investigating a conspecific’s carcass.

In primates and cetaceans^2^, individuals have been observed physically supporting and/or carrying a deceased conspecific (usually a young animal) for periods ranging from hours to more than a month^3^. Seeing the corpse might be useful for an animal as it might learn about death from specific features, including the total lack of responsiveness or animacy^4^. Indeed, different animal species exhibit complex responses towards their dead^5^, probably, not only as a consequence of several sensory characteristics but also from changes in biological motion perception^6^.

The ability to mourn has been suggested for a variety of other animal species, including dogs^7^, but evidence is currently sparse and numerous limitations have to be considered, including the risk of interpreting anthropocentrically and the difficulty to design replicable and representative scientific experiments. If seeing the corpse is part of the death ritual, considering that domestic dogs have no access or only a brief access to the corpse, then only the reaction to separation from the bonded individual might be evaluated. From a biological perspective, exhibiting patterns of grief-like behaviour in dogs could be seen as a response to separation^8^ from an attachment figure^9–11^.

According to Bekoff^12^, dogs might display grief as a result of a close relationship, due to their highly social nature. However, behavioural responses towards dying/dead conspecifics have been only rarely observed in wild canids^13,14^. Boyd et al.^14^ briefly presented evidence of wild wolves (*Canis lupus*) burying the carcasses of two-week old pups. Appleby^13^ described the death of a three-month old dingo pup and the associated responses of the pup’s mother and fellow littermates: the deceased pup was transported to different locations in the days following its death^13^. As for pet dogs, there is a long history of anecdotal reports by owners about individuals grieving over the loss of a companion conspecific^15^; however, few scientific data have been published documenting grief-like behavioural reactions in domestic dogs^15^. Even the definition of grief and the “ability to mourn” in dogs is not straightforward and the same difficulties exist in relation to humans, especially young children. It is worth mentioning that at some levels the mind of a dog is equivalent to that of a human child between two and three years of age^16^. Moreover, only if the exposure to the body resulted in a significant difference compared to “simply” separation stress, then we might start talking about a grief-like reaction. Children in the age range of two to five years might not have the concept of death, but the concept of loss of a caregiver is likely to trigger the pattern of behaviours that we speak of as representing grief and mourning. Dogs do form emotional bonds which may include companion animals in their household, and hence removing that companion can be expected to cause behavioural changes which certainly overlap those behaviours that we normally interpret as being grief and mourning.

Therefore, the aim of this study was to identify and quantify grief-related reactions over the loss of a companion dog in an Italian pet dog population. For this purpose, we used a previously scientifically validated questionnaire (the Mourning Dog Questionnaire-MDQ)^17^ designed to obtain data on grief responses in dogs and dog owners after the loss of a pet dog. Dog owners may have a tendency to over-report canine emotions of grief^18^, which can lead them to inflate reactions of dogs as a reflection of their own pet loss-related bereavement. There are also changes in the daily routine and perhaps the attitudes and responses of the owner of the deceased dog, and these may affect the behaviour of the survivor as well. Considering and cross-referencing owners' reports on both themselves and their dog partner’s is a unique aspect of the current study, that allows the assessment of the risk of biased responding.

In particular, we aimed to test the hypothesis that if any behavioural changes were observed by the owner in a dog after the loss of a companion dog, these reflected real behavioural changes presumably resulting from the loss of the conspecific, regardless of the owner’s own feelings and memories over the same loss.

The particular structure of the questionnaire used also enabled us to pursue a secondary aim, namely to investigate whether, based on this species’ high sociality^19^, a pet dog’s grief-like reactions may be affected by the quality of the relationship between the two animals or by the response to having seen the corpse. Based on the literature related to attachment and separation, we hypothesised that behaviours referable to grief would be detected in survivor dogs, at least if they had had a long lasting and affiliative relationship with the deceased one. Evidence of the expression of such a complex social emotion by pet dogs may be important to considerations of animal welfare, given the large number of multi-dog households^20^.

## Materials and methods

### Procedure

We used an owner-answered online questionnaire to study both owner and surviving dog reactions after the loss of a cohabiting dog and collected a cross-sectional convenience sample of Italian pet dogs. Here we present results concerning the surviving dogs.

Since the study is part of a larger project, the first step consisted of the statistical validation process of the Italian version of the MDQ, followed by an analysis of correlations among all the constructs and between the constructs and participant/pet characteristics. There was not a specific timeframe, but the owners had to report when the animal passed away. The study was approved by the University of Padua Ethics Committee (46DF1164A03D63129CEE38D7571F8FB7). All methods were performed in accordance with the relevant guidelines and regulations of our institutions. Participants were given written information about the aim and the procedures of the study and the right to withdraw at any time. For ethical reasons, participants were only informed that the survey was part of a research study that aims to explore whether and how a dog and his/her owner may experience the loss of a companion dog.

In addition, they were assured that the survey was anonymous, and confidentiality would be maintained by the researchers. Before data collection, written informed consent was obtained from each participant. Participation was voluntary.

### Participants

The survey was published on the internet and social networks (e.g., Facebook), targeting Italian participants who were older than 18 years and had experienced the death of a dog. Moreover, to meet key requirements for enrolment of a larger project of which this study is a part, all the recruited dog owners had at least two dogs at the time of the dog’s death.

### Materials

The questionnaire and its validation have been described in full in Uccheddu et al.^17^ The questionnaire (see Appendix A) composed of three parts. The first part is divided into two sections: (1) owner demographics, including gender, age, educational level, marital status, household composition and presence of children, occupational status; (2) information on the deceased dog, including age, sex, time since dog’s death, length of ownership, cause of death, and whether the owner lived alone at the time of the death. (3) Moreover, we enquired about the relationship between the two dogs in terms of being: friendly, agonistic, mutual tolerance, parental based on both owner observation and genetic association. The dimensions were assessed with a 5-point Likert scale (from strongly disagree to strongly agree) based on their point of view. Then, the survey targeted change during the current life of the surviving dog’s behaviours (Playing, Sleeping, Eating, Fear, Vocalisation, Elimination, Attention seeking, Level of activity, Other) and duration of the variation, if any. The questionnaire also included a comprehensive background section, consisting of questions dealing with the shared items before loss (Food, Resting area, Objects/toys, None, evaluation of each item as presence or absence), sharing activities between two dogs (evaluation of each item on a 3-point Likert scale from never to often) in terms of sharing activities sleeping, sharing activities fighting, sharing activities grooming each other and sharing activities playing, behavioural changes in surviving dog after the death of the other dog (evaluation of each item as presence or absence) in terms of playing less, eating less, level of activity reduced, sleeping more, fearfulness increased, vocalization increased and attention seeking increased and duration of the behavioural alteration.

The second part is composed of a combination of five established questionnaires, namely the Pet Bereavement Questionnaire (PBQ)^21^, the Lexington Attachment to Pets Scale (LAPS)^22^, the Animal-Human Continuity Scale (AHCS)^23^, the Positivity Scale (P-Scale)^24^ and the Testoni Death Representation Scale (TDRS)^25^, which, individually, have already been demonstrated to have good internal reliability. The Italian versions of PBQ, LAPS and TDRS have already demonstrated good factor structure and good construct validity **(**the degree to which all questionnaires are measuring appropriately)^25^. All instruments, except for TDRS, have been forward- and backward-translated by two independent translators^26^.

Briefly, the PBQ is a 16-item 4-point Likert-type scale which assesses pet bereavement distress. The PBQ is composed of three distinct factors: Grief, Anger and Guilt. The LAPS is a 23-item scale measuring pet attachment related to each of the following factors: General Attachment, Animals Substituting People and Animal Rights/Animal Welfare. The AHCS is a 12-item 7-point Likert-type animal attitude scale that measures the extent to which the respondents view humans and animals as on the same continuum or in a dichotomous fashion. The scale is composed of three factors: Rational Capacity, Superiority versus Equality, and Evolutionary Continuum. The P-Scale is an 8-item unidimensional 5-point Likert type scale designed to measure positivity, that is, the tendency to view life and experiences with a positive outlook. The TDRS is a 6-item self-report measure which assesses the attitudes of individuals toward the ontological representation of death as a passage to an afterlife or as a form of annihilation.

### Statistical analysis

After the check of normality distribution for continuous variables by Shapiro–Wilk (p < 0.001 for all variables except for PBQ total score), bivariate correlations between the construct scales and the characteristics of the participants and the characteristics of the companion animals was initially examined using Spearman’s rho coefficient; Mann–Whitney Tests were used where the interest was in differences in behavioural changes. Values of p < 0.05 were considered significant in this preliminary assessment. Association with changes in the surviving dog’s behavior and all dog’s and owner’s characteristics was also evaluated from initial bivariate correlations. Significant variables from the bivariate analysis (the bond between the two dogs, shared items and activities, PBQ) were entered into a multiple logistic regression model. The strength of the associations was expressed as the odds ratio (OR) and 95% confidence interval (95%CI), p-values < 0.05 were considered significant. Analyses were carried out using SPSS 24 (IBM Corp. Released 2016. IBM SPSS Statistics for Windows, Version 24.0. Armonk, NYproducer).

## Results

### Demographics

We collected reports from 426 Italian dog owners (384 females and 42 males) with a mean age of 42.19 ± 11.06 (SD) years (range 18.00–70.00).

When the dog died, 12.90% of respondents were living alone, 66.40% of participants reported having lost their dog over one year before, 21.70% less than 6 months before, and 11.90% between 6 and 12 months before participating in the survey. The average length of the deceased dog’s ownership was 9.81 ± 4.45 (SD) years (range 0.20–20.00); the dog’s mean age at death was 11.55 ± 3.81 (SD) years (range 0.50–20.00). With respect to the circumstances of the dog’s death, 52.10% of the respondents declared that it was unexpected, while 57.2% had opted for euthanasia for a health reason, 0.2% for a behavioural reason.

About 92.5% of participants reported a duration greater than 12 months of the time the two dogs had lived together. Their relationship was described as friendly for 69% of owners, non-agonistic for 56% of owners, mutual tolerance for 56% of owners and parental for 48% of owners. The two dogs shared several activities such as sleeping (66%), no fighting (54%), grooming each other (27%), playing (49%) but they also shared food (36%), resting area (86%), objects/toys (58%) and only 9% shared nothing.

### Canine behavioural changes

Several negative behavioural changes were commonly reported in the surviving dog after the death of the other dog: attention seeking increased (67%), playing less (57%), level of activity reduced (46%), sleeping more (35%), fearfulness increased (35%), eating less (32%) and vocalisation increased (30%).

When any behavioural alteration was observed, 24.9% of the owners observed it for more than 6 months, 32.2% between 2 and 6 months, 29.4% for less than 2 months. No behavioural changes were observed by 13.4% of the owners. Animal’s sex (r = 0.048; p = 0.324), neuter status (r = 0.069; p = 0.153), age at death (r = 0.009; p = 0.856) and breed (r = 0.030; p = 0.541), but also viewing of the corpse (r = 0.028; p = 0.570), did not affect the duration of behavioural alterations, when they occurred. Table 1 shows the correlations between relationship-related characteristics and behavioural changes observed in the surviving dog after the death of the other dog. In particular, it can be noted that the duration of the relationship between the two dogs positively correlated with “playing less”, “level of activity reduced”, “sleeping more” but no correlation was found with variables such as “fearfulness increased”, “vocalisation increased”, “attention seeking increased” and “duration of the behavioural alteration”. Also, a friendly and parental relationship between the two dogs was associated with stronger behavioural changes, while no association was found between behavioural variables and an agonistic/mutual tolerance relationship. Associations between characteristics of the relationship, constructs related to owner and behavioural change observed in the surviving dog after the death of the other dog are reported in Supplementary Information.

**Table 1:** Correlation between characteristics of the relationship between the two dogs and behavioural changes observed in the surviving dog after the death of the other dog.

	Playing less	Eating less	Level of activity reduced		Sleeping more	Fearfulness increased	Vocalisation increased	Attention seeking increased		Duration of the behavioural alteration	
Duration of the relationship	0.169**	0.047	0.172**		0.096*	0.018	0.067	0.084		0.084	
Type of relationship-friendly	0.214**	0.039	0.157**		0.137**	−0.005	0.004	0.152**		0.158**	
Type of relationship-agonistic	0.009	−0.046	−0.007		−0.019	0.023	0.015	0.095		−0.012	
Type of relationship-mutual tolerance	−0.077	0.051	0.033		0.039	0.075	0.008	0.026		0.026	
Type of relationship-parental	0.163**	0.188**	0.150**		0.091	0.166**	0.095*	0.121*		0.201**	

*p < 0.05; **p < 0.01; ***p < 0.001.

### MDQ and behavioural changes

In Table 2, correlations between MDQ parts (PBQ, Laps, AHCS and P-scale scores) and behavioural changes of the surviving dog are reported. “Grief” as assessed by PBQ positively correlated with “eating less” and “fearfulness increased”. “PBQ Anger” and “PBQ Total” positively correlated with “fearfulness increased”. Except for the positive correlation between P scale and playing less, no other correlations were revealed between constructs related to the owner and behavioural change in surviving dogs.

**Table 2:** Correlation between constructs related to owner and behavioural change observed in the surviving dog after the death of the other dog.

	Playing less	Eating less	Level of activity reduced	Sleeping more	Fearfulness increased	Vocalisation increased	Attention seeking increased	Duration of the behavioural alteration
PBQ_Grief	0.081	0.122*	0.082	0.037	0.145**	0.063	0.095	−0.021
PBQ_Anger	−0.022	−0.022	-0.008	0.060	0.174**	0.006	−0.008	0.085
PBQ_Guilt	−0.018	−0.002	−0.067	0.065	0.019	−0.025	−0.034	0.014
PBQ_Total	0.015	0.057	−0.001	0.057	0.147**	0.024	0.040	0.025
Laps_General Attachment	0.088	0.074	0.098	0.030	0.020	0.088	0.056	0.080
Laps_People Substituting	−0.010	0.087	0.002	−0.032	0.019	0.064	0.073	0.012
Laps_Animal Rights	−0.026	0.093	0.003	−0.002	0.011	0.004	0.018	−0.012
Laps_Total	0.027	0.098	0.044	−0.004	0.025	0.075	0.073	0.040
AHCS_Total	−0.004	0.098	0.003	−0.044	0.027	0.017	−0.031	0.043
P_Scale	0.112*	0.062	0.079	0.064	-0.095	-0.012	0.051	0.029

*p < 0.05; **p < 0.01; ***p < 0.001.

### Shared items and activities between the two dogs and behavioural change

In Table 3, correlations between shared items between the two dogs and behavioural change observed in the surviving dog after the death of the other dog are reported. In Table 4, correlations between behavioural changes observed in the surviving dog after the death of the other dog and shared activities between the two dogs are reported. These tables indicated that the sharing of items or activities is positively correlated with behavioural changes, in particular with a reduction in the level of activity, while no sharing is negatively correlated with all the observed behavioural changes.

**Table 3:** Correlation between shared items between the two dogs and behavioural change observed in the surviving dog after the death of the other dog.

	Playing less	Eating less	Level of activity reduced	Sleeping more	Fearfulness increased	Vocalisation increased	Attention seeking increased	Duration of the behavioural alteration
Sharing_food	0.126**	0.174**	0.136**	0.155**	−0.009	−0.015	−0.048	−0.004
Sharing_resting area	0.132**	0.124*	0.174**	0.128**	0.058	0.075	0.070	0.131**
Sharing_objects/toys	0.264**	0.051	0.194**	0.112*	0.002	0.035	0.108*	0.136**
Sharing_none	−0.201**	−0.132**	−0.210**	−0.128**	−0.025	−0.063	−0.158**	−0.143**

*p < 0.05; **p < 0.01; ***p < 0.001.

**Table 4:** Correlation between shared activities between the two dogs and behavioural change observed in the surviving dog after the death of the other dog.

	Playing less	Eating less	Level of activity reduced	Sleeping more	Fearfulness increased	Vocalisation increased	Attention seeking increased	Duration of the behavioural alteration
Sharing activities_sleeping	0.203**	0.081	0.117*	0.054	0.031	0.036	0.048	0.119*
Sharing activities_fighting	0.115*	0.055	0.045	0.056	0.024	0.005	−0.035	0.106
Sharing activities_grooming each other	0.187**	0.132**	0.124*	0.134**	0.008	0.030	0.022	0.108*
Sharing activities_playing	0.351**	0.092	0.213**	0.129**	0.006	0.046	0.089	0.147**

*p < 0.05; **p < 0.01; ***p < 0.001.

### Time since the death

Time since the death of the dog negatively correlated with PBQ Grief (r = -0.141, p = 0.01) and PBQ-Total (r = -0.100, p = 0.05), and positively with P scale (r = 0.098, p = 0.05). No correlation was found with PBQ_Anger and PBQ Guilt, LAPS General Attachment, LAPS_People Substituting, LAPS_Animal Rights, LAPS Total, AHCS Total.

### Predicting surviving dog behaviour

Multiple logistic regression model results are summarised in Table 5, where only statistically significant factors are reported. The single significant predictor for playing less was the friendly relationship between the two dogs; three significant predictors for eating less were the friendly or parental relationship between the two dogs and owner’s grief; one predictor for level of activity reduce and sleeping more given by sharing food; two predictors for increased fearfulness were a parental relationship between the two dogs and owner’s anger; the single predictor for increased vocalization was a parental relationship between the two dogs and one predictor for increased attention seeking was owner’s grief.

**Table 5:** Multiple logistic regression model predicting dog behaviour’s changes.

Predictive factors	B	S.E.	Sig	EXP(B)	95% CI for EXP(B)	
					Lower	Upper
**Playing less**						
Relationship between the two dogs (friendly)	0.265	0.108	0.014	1.303	1.055	1.61
**Eating less**						
Relationship between the two dogs (friendly)	-0.246	0.12	0.041	0.782	0.681	0.99
Relationship between the two dogs (parental)	0.343	0.095	<0.001	1.409	0.213	0.129
PBQ_grief	0.075	0.028	0.008	1.077	1.019	1.139
**Level of activity reduced**						
Sharing_food	0.455	0.21	0.030	1.576	1.044	2.378
**Sleeping more**						
Sharing_food	0.599	0.216	0.006	1.821	1.192	2.782
**Fearfulness increased**						
Relationship between the two dogs (parental)	0.362	0.094	< 0.001	1.436	1.194	1.727
PBQ_Anger	0.112	0.043	0.009	1.119	1.029	1.216
**Vocalisation increased**						
Relationship between the two dogs (parental)	0.221	0.092	0.017	1.247	1.040	1.494
**Attention seeking increased**						
PBQ_grief	0.058	0.026	0.027	1.060	1.007	1.115

*B* regression coefficient, *S.E.* standard error, *Sig.* significance, *EXP(B*) exponentiation of the B coefficient (odds ratio), *CI* confidence interval.

## Discussion

Dog owners reported several statistically significant changes in the surviving dog after the death of the companion dog, both in terms of activities (“playing”, “sleeping”, and “eating”) and emotions (fearfulness), which occurred as a function of the quality of the relationship between the two animals. By contrast, the time the two dogs had spent together had no effect. This highlights the importance of several controls put in place to consider the risk of confounds resulting in reporting bias the owners. When the relationship was rated as friendly (versus agonistic or mutual tolerance), the surviving dog was significantly more likely (1.3 times) to play less and to eat more or similar after the death event. Emotional eating, or changes in eating behaviour due to negative emotions, has been reported in dogs^27^. A parental relationship (defined as parent–offspring relationship) was a significant predictive factor for reduction in eating of the surviving dog. Dogs living in the same household might develop a strong affiliative bond, regardless of whether they have a kinship relationship^28^. From an ecological point of view, both affiliative and parental bonds are important components of the natural social organisation of free ranging dogs. Packs of free-ranging dogs are often composed of relatives^29^, who are expected to establish close family relationships. Social animals have a strong tendency to co-operate and synchronise their behaviour^30^, and this happens in domestic dogs as well^31^. This coordination of group activities is fundamental in order to maintain group cohesion and eventually allow animals to get the benefits of social living^32^, and may be disrupted in the case of a death in the group. Thus, a strong affiliation during life may have led pet dog dyads in our study to integrate their routines, which may explain the changes observed after the death event in the behaviours of surviving dogs. In support of this hypothesis we found that if dogs used to share food during life, the surviving dog was more likely to reduce her/his level of activities and sleep more after the loss. There is plenty of literature on emotional bonds (see for example^10,33,34^) among dogs, but the form the bond takes is less clear. For this reason, although attachment can be seen as describing affectionate bonds, here we referred to dog–dog attachment as one of several elements to the emotional bond but focused on the provision of safety and security.

Alternatively, nonhuman animals’ capacity for grief might be explained by reference to attachment theory or social support and the consequent separation distress^35^. Indeed, previous research has shown that characteristics of the relationship between two dogs can affect their behaviour in a Ainsworth Strange Situation Test^28^. Attachment in social species, such as dogs, is important for survival^35^, and to a certain extent is related to the acquisition of ethological (social) and ecological (food, etc.) skills^36^. Indeed, attachment in a broad sense^37^, through its role in shaping future close social relationships, may also be considered to be the basic organisational factor for any species' social structure leading to group bonds^38^. Loss can be considered an interruption of the attachment bond^15^, which, from an ecological perspective, can explain the impact it has on the behaviours of a surviving individual.

Bowlby’s attachment theory has been evaluated in the human–dog relationship^39^: the attachment bond between a caregiver and a dog has been cited as an explanation for the strength of the human–pet bond and the intensity of pet loss in humans^17^; our results indicate that it could also affect any canine survivor’s behaviour as well. Importantly our study advances those conducted previously on pet dog grief in that it investigates both perceived canine and human grief-like behaviours. Surprisingly, in our study dog-owner attachment, the owner's vision of life, humanisation of pets and the view of animals and humans as being on the same continuum, rather than being separated entities (as assessed by LAPS, AHCS, Positivity and TDRS questionnaires) did not correlate with any reported canine behavioural changes occurring after the conspecific died. This is important because it indicates that the owner is not simply projecting grief on their dog based on their own sentiments; the reported changes are thus more likely to be real. However, a reduction in the surviving dog’s food intake is significantly more likely to occur in case of grief of the owner (PBQ Grief). It might be that when surviving dogs live with owners that answered with highest scores in PBQ Grief items (such as “I am very upset about my pet’s death”), there might be some form of emotional contagion; considered the “clear evidence of a primitive form of cross-species empathy” by some authors^40^. Such emotional contagion may be the result of a long history of affiliation with humans, as it is seen as a fundamental feature of animals that live in close social groups^41^. Another explanation for the dogs’ behaviour toward distressed owners is that dogs feel negative emotion and so seek comfort or relief from distress^42^. Social animals, spending time together, are continuously exposed to shared stressors which could affect different individuals similarly^43^. Dogs acquire an ability to respond appropriately to human communicative gestures and facial expressions, referring to human information, especially their emotional expression in their decision-making^44^.

A change in food intake in dogs after the loss of a conspecific has been reported previously by Walker^45^ (83% of dogs displayed a reduction in the amount of food consumed) and Schultz^46^ (36% of dogs reduced consumption). However, according to our results, owners' perceptions regarding the variation of this behaviour are at least partially influenced by their own emotional state. Owner perceived-emotional eating in companion dogs might also be seen as an expression of affection and attachment^47^. In terms of dog emotions, changes were reported by the owners with respect to fear, with these changes appearing to be affected by the owner’s own emotional state. As assessed using the PBQ scale, in fact, the level of fear in the surviving dog was positively correlated with owners' level of suffering, anger and psychological trauma. This could be the product of either owner’s interpretation and/or genuine changes in behaviour. Emotions are part of an adaptive coping strategy with the circumstances that have elicited them^48^. In social species, they are often acquired indirectly through social transmission^49^, and occur also interspecifically, between dogs and humans^50^. The sight, sound or smell of a scared individual, whether it is another dog or the owner, within an interspecific social group, may trigger fear responses in others^49^. Recently, D’Aniello et al^51^ showed how human body odours (chemosignals) produced under emotional conditions of fear provide information that is detectable by pet dogs, ultimately affecting their physiological and emotional status and, consequently, their behaviours^52^. Of course, we do not know if surviving dogs in our study were able to understand that an owner’s suffering, anger and psychological trauma revealed by PBQ resulted from the feeling of loss. However, our data suggest that these three human reactions had an impact on the reported level of fear in the surviving dog.

Three scenarios may be used to interpret the result. Firstly, we can hypothesise that a perception of owner suffering or anger might trigger fear in the surviving dog. This is supported by social-referencing studies reporting that dogs modify their behaviour in accordance with their owner’s positive or negative emotional reaction^44,53^; this may depend on their social ties, as previously shown in non-human primates^54^. Secondly, the owner’s suffering, anger and psychological trauma may have affected how they perceived their dog’s emotional status. Another intriguing result is that the surviving dogs showed no difference in behaviour depending on whether or not they had seen the body of the deceased dog. Our results seem in line with other companion dog reports^15^ in which 58% of surviving dogs viewed their deceased companion’s body^15^. Although we did not specifically investigate the behaviour shown by dogs in front of the corpse, Walker^15^ reported that, upon viewing the deceased animal, 73% dogs were reported to sniff and investigate the body of a dead canine companion, while only a small number did not show any interest. In other species, particularly some considered to have very high cognitive abilities, such as elephants, behavioural interactions with carcasses of conspecifics have been reported^55^. Elephants showed approach and exploratory (sniffing and inspecting) behaviours^56^. Cetaceans and primates have also been observed engaged in complex rituals^55^. Chemical cues that trigger dead body exploration could be the reason for an animal showing interest towards a dying conspecific^3^. Thirdly from a survival perspective: the loss of, or dead conspecifics can be a relevant source of information regarding potential risk in an area, being a trigger for subsequent risk-reducing behavioural modification^57^. Moreover, when faced with danger, animals like primates involved in loss can seek support from others and significantly improve their ability to deal with threat through effective cooperation^58^. A surviving dog may perceive a potential threat deriving from a companion dog’s death and seek the owner’s help to deal with this situation; if the owner herself is angry/ grieving, she/he may be less able to give the dogs the help they are seeking, resulting in increased anxiety and fear in the dog. Finally, owner’s attitudes and behaviours changes after the death of the dog might be important factors in the surviving dog’s behaviour due to expecting or anticipating events which were no longer occurring. As widely reported in literature, changing dog’s routines, even unintentionally, could result in anxiety or frustration^59^.

In our survey, we enquired about specific individual items to avoid the risk that owners’ reports were conditioned by memory or by suffering that tended to diminish over time. Memory for facts and events typically becomes less accessible over time^60^. It is thus possible to reduce or suppress the false recognition effect of memories when participants are encouraged to focus on distinctive properties of individual items^60^. Importantly in our study, the time that passed since the dog’s death at the time of the survey did not affect the different behavioural changes reported in the surviving dog.

However, since human–dog bonding can have an effect on a dog’s perception of a dead conspecific, it would be difficult attributing a specific pattern, if any, of exploration. Not only anthropomorphism may play a role in attributing a specific function to the dogs’ behaviour, but attention to a deceased individual might also occur as a result of the owners’ increasing attention (stimulus enhancement)^61^. Not surprisingly, an emotional contagion might also be considered, since stress seems contagious between dogs and owners^43^. Our results might suggest that the dogs are responding to the “loss “of an affiliate, more than their “death” per se*.*

## Conclusion

Behavioural changes observed, and their association with the dogs’ relationship and dogs’ social bonds, might be indicative of separation stress after loss. The duration of time that the dogs lived together is not predictive of the behavioural effects (a relationship which one might expect if the results were simply a matter of disruption of the dog’s daily routine because their housemate has passed away). This is potentially a major welfare issue that has been overlooked, considering the relatively high number of dogs who live with at least another companion dog^62^ and the dog aging population^63^; so they are therefore at high risk of experiencing the loss of a close conspecific. The understanding of behavioural patterns after loss in non-human animals can be helpful in recognising these animals’ emotional needs. However, even if we recognise the importance of these results, we still cannot confirm it was grief. More research is clearly needed (Supplementary Information).

## Supplementary Information

Supplementary Information.
