# Supplementary material for: Domestic dogs (Canis familiaris) grieve over the loss of a conspecific
Source: Sci Rep. 2022 Feb 24;12:1920. doi: 10.1038/s41598-022-05669-y (PMC8873218; doi:10.1038/s41598-022-05669-y)
Supplement: Supplementary file 1 — Supplementary Information. [file 41598_2022_5669_MOESM1_ESM.docx]

Supplementary material

Association between characteristics of the relationship, constructs related to owner and behavioural change observed in the surviving dog after the death of the other dog (average ranks for behavioural change presence/not presence - Mann-Whitney Test)

|  | Playing less | | | | | | Eating less | | | | Level of activity reduced | | | | | | **Sleeping more** | | | | | | | Fearfulness increased | | | | | | | | Vocalisation increased | | | | | | | Attention seeking increased | | | | | | |  |
| --- | --- | --- | --- | --- | --- | --- | --- | --- | --- | --- | --- | --- | --- | --- | --- | --- | --- | --- | --- | --- | --- | --- | --- | --- | --- | --- | --- | --- | --- | --- | --- | --- | --- | --- | --- | --- | --- | --- | --- | --- | --- | --- | --- | --- | --- | --- |
| Variables | no | | yes | | test MW | | no | yes | test MW | | no | yes | | test MW | | No | | | yes | | test MW | | no | | | yes | | test MW | | | no | | yes | | test MW | | | no | | yes | | test MW | | |  |  |
| Duration of relationship |  | |  | |  | |  |  |  | |  |  | |  | |  | | |  | |  | |  | | |  | |  | | |  | |  | |  | | |  | |  | |  | | |  |  |
| Duration of the relationship -dogs | 203,57 | | 222,74 | | -3.48*** | | 212,69 | 218,30 | -0,96 | | 205,61 | 225,02 | | -3.55*** | | 210,57 | | | 221,94 | | -1.98* | | 213,78 | | | 215,87 | | -0,37 | | | 212,06 | | 220,27 | | -1,38 | | | 207,75 | | 217,82 | | -1,74 | | |  |  |
| Duration of the relationship Decaesed Dog-owner | 215,19 | | 213,10 | | -0,17 | | 218,22 | 205,16 | -1,02 | | 221,95 | 204,63 | | -1,45 | | 218,17 | | | 206,14 | | -0,96 | | 214,09 | | | 213,82 | | -0,02 | | | 211,29 | | 220,40 | | -0,70 | | | 205,42 | | 218,18 | | -1,01 | | |  |  |
| Duration of the relationship Surviving Dog-owner | 208,33 | | 219,15 | | -2.58** | | 214,60 | 214,28 | -0,07 | | 211,51 | 218,04 | | -1,56 | | 212,75 | | | 217,81 | | -1,16 | | 215,13 | | | 213,30 | | -0,42 | | | 212,86 | | 218,39 | | -1,21 | | | 208,32 | | 217,54 | | -2.08* | | |  |  |
| Time passed after the death of the dog | 215,45 | | 213,78 | | -0,17 | | 213,10 | 217,44 | -0,41 | | 210,23 | 219,56 | | -0,93 | | 220,57 | | | 203,02 | | -1,67 | | 213,47 | | | 216,45 | | -0,28 | | | 218,17 | | 205,80 | | -1,13 | | | 213,82 | | 214,84 | | -0,10 | | |  |  |
| Relationship between the two dogs |  | |  | |  | |  |  |  | |  |  | |  | |  | | |  | |  | |  | | |  | |  | | |  | |  | |  | | |  | |  | |  | | |  |  |
| Friendly | 186,42 | | 235,67 | | -4.41*** | | 211,40 | 221,01 | -0,81 | | 198,02 | 234,01 | | -3.24** | | 203,11 | | | 236,05 | | -2.84** | | 214,94 | | | 213,67 | | -0,11 | | | 214,20 | | 215,21 | | -0,08 | | | 189,67 | | 226,70 | | -3.15** | | |  |  |
| Agonistic | 213,28 | | 215,42 | | -0,18 | | 218,27 | 206,57 | -0,95 | | 215,31 | 213,54 | | -0,15 | | 216,11 | | | 211,45 | | -0,38 | | 212,51 | | | 218,27 | | -0,47 | | | 213,30 | | 217,33 | | -0,32 | | | 198,39 | | 222,41 | | -1,95 | | |  |  |
| Mutual Tolerance | 225,16 | | 206,46 | | -1,59 | | 210,31 | 223,31 | -1,04 | | 210,89 | 218,78 | | -0,68 | | 211,13 | | | 220,88 | | -0,80 | | 207,94 | | | 226,91 | | -1,55 | | | 213,91 | | 215,89 | | -0,16 | | | 210,00 | | 216,71 | | -0,54 | | |  |  |
| Parental | 191,93 | | 231,52 | | -3.37** | | 198,95 | 247,17 | -3.88*** | | 197,96 | 234,08 | | -3.10** | | 206,54 | | | 229,56 | | -1,88 | | 199,99 | | | 241,94 | | -3.43** | | | 207,06 | | 232,12 | | -1.97* | | | 193,75 | | 224,69 | | -2.50* | | |  |  |
| Shared activities |  | |  | |  | |  |  |  | |  |  | |  | |  | | |  | |  | |  | | |  | |  | | |  | |  | |  | | |  | |  | |  | | |  |  |
| Sleeping | 190,48 | | 232,61 | | -4.19*** | | 208,72 | 226,64 | -1,68 | | 203,47 | 227,56 | | -2.41* | | 210,43 | | | 222,20 | | -1,12 | | 212,19 | | | 218,88 | | -0,64 | | | 212,08 | | 220,22 | | -0,75 | | | 207,38 | | 218,00 | | -1,00 | | |  |  |
| Fighting | 199,70 | | 225,66 | | -2.39* | | 210,29 | 223,35 | -1,13 | | 209,88 | 219,97 | | -0,93 | | 209,96 | | | 223,08 | | -1,16 | | 212,56 | | | 218,18 | | -0,50 | | | 214,12 | | 215,41 | | -0,11 | | | 220,00 | | 211,80 | | -0,72 | | |  |  |
| Grooming | 189,50 | | 233,35 | | -3.86*** | | 203,90 | 236,77 | -2.73** | | 201,31 | 230,11 | | -2.55* | | 203,22 | | | 235,85 | | -2.76** | | 213,86 | | | 215,72 | | -0,16 | | | 212,23 | | 219,88 | | -0,62 | | | 210,88 | | 216,28 | | -0,45 | | |  |  |
| Playing | 169,06 | | 248,77 | | -7.26*** | | 207,40 | 229,42 | -1,89 | | 192,49 | 240,55 | | -4.40*** | | 203,97 | | | 234,42 | | -2.66** | | 214,05 | | | 215,35 | | -0,11 | | | 211,16 | | 222,42 | | -0,95 | | | 200,16 | | 221,55 | | -1,85 | | |  |  |
| Owner grief |  | |  | |  | |  |  |  | |  |  | |  | |  | | |  | |  | |  | | |  | |  | | |  | |  | |  | | |  | |  | |  | | |  |  |
| PBQ_Grief | 203,02 | | 223,16 | | -1,67 | | 204,16 | 236,24 | -2.52* | | 205,22 | 225,48 | | -1,69 | | 211,18 | | | 220,78 | | -0,77 | | 201,49 | | | 239,10 | | -3.00** | | | 209,48 | | 226,41 | | -1,30 | | | 197,86 | | 222,67 | | -1.96* | | |  |  |
| PBQ_Anger | 217,62 | | 212,15 | | -0,46 | | 216,35 | 210,62 | -0,45 | | 215,37 | 213,47 | | -0,16 | | 209,12 | | | 224,68 | | -1,25 | | 199,01 | | | 243,81 | | -3.59*** | | | 214,01 | | 215,66 | | -0,13 | | | 215,94 | | 213,79 | | -0,17 | | |  |  |
| PBQ_Guilt | 217,01 | | 212,61 | | -0,37 | | 214,63 | 214,22 | -0,03 | | 222,04 | 205,58 | | -1,38 | | 208,67 | | | 225,52 | | -1,35 | | 212,76 | | | 217,79 | | -0,40 | | | 216,53 | | 209,70 | | -0,52 | | | 220,45 | | 211,58 | | -0,70 | | |  |  |
| PBQ_Total | 212,40 | | 216,08 | | -0,31 | | 209,68 | 224,62 | -1,17 | | 214,59 | 214,39 | | -0,02 | | 209,35 | | | 224,25 | | -1,19 | | 201,32 | | | 239,43 | | -3.03*** | | | 212,56 | | 219,11 | | -0,50 | | | 207,51 | | 217,94 | | -0,82 | | |  |  |
| laps_GeneralAttachment | 202,09 | | 223,86 | | -1,81 | | 208,23 | 227,68 | -1,53 | | 203,50 | 227,53 | | -2.01* | | 211,80 | | | 219,61 | | -0,63 | | 212,69 | | | 217,92 | | -0,42 | | | 207,49 | | 231,13 | | -1,82 | | | 204,66 | | 219,34 | | -1,16 | | |  |  |
| laps_PeopleSubstituting | 215,94 | | 213,42 | | -0,21 | | 207,09 | 230,08 | -1,80 | | 214,31 | 214,73 | | -0,03 | | 217,34 | | | 209,13 | | -0,66 | | 212,78 | | | 217,75 | | -0,40 | | | 209,34 | | 226,73 | | -1,33 | | | 201,67 | | 220,80 | | -1,51 | | |  |  |
| laps_AnimalRights | 218,04 | | 211,83 | | -0,54 | | 206,97 | 230,32 | -1,93 | | 214,13 | 214,94 | | -0,07 | | 214,64 | | | 214,23 | | -0,04 | | 213,59 | | | 216,23 | | -0,22 | | | 214,17 | | 215,28 | | -0,09 | | | 211,52 | | 215,96 | | -0,37 | | |  |  |
| laps_Total | 210,68 | | 217,38 | | -0,55 | | 206,14 | 232,06 | -2.03* | | 209,53 | 220,38 | | -0,90 | | 214,86 | | | 213,82 | | -0,08 | | 212,28 | | | 218,70 | | -0,51 | | | 208,47 | | 228,80 | | -1,55 | | | 201,56 | | 220,86 | | -1,52 | | |  |  |
| AHCS_Total | 215,07 | | 214,07 | | -0,08 | | 206,11 | 232,12 | -2.04* | | 214,14 | 214,92 | | -0,07 | | 218,49 | | | 206,95 | | -0,92 | | 212,06 | | | 219,11 | | -0,56 | | | 213,12 | | 217,77 | | -0,36 | | | 219,89 | | 211,85 | | -0,63 | | |  |  |
| P_Scale | 198,53 | | 226,54 | | -2.32* | | 209,24 | 225,55 | -1,28 | | 205,59 | 225,05 | | -1,62 | | 208,75 | | | 225,38 | | -1,32 | | 222,98 | | | 198,46 | | -1,95 | | | 215,46 | | 212,22 | | -0,25 | | | 205,49 | | 218,93 | | -1,06 | | |  |  |
| * *p* < 0.05; ** *p* < 0.01; *** *p* < 0.001 | |  | |  | |  | |  |  |  | | |  | |  | | |  | |  | |  | | |  | |  | |  |  | | | |  | |  |  | | | |  | |  |  | | |
